# High-Efficiency Radiation via Fast Electron Beam Pinching in Nonuniform Plasmas

**DOI:** 10.34133/research.1330

**Published:** 2026-06-19

**Authors:** Xing-Long Zhu, Min Chen, Zheng-Ming Sheng

**Affiliations:** ^1^ Institute for Fusion Theory and Simulation, School of Physics, Zhejiang University, Hangzhou 310058, China.; ^2^ State Key Laboratory of Dark Matter Physics, School of Physics and Astronomy, Shanghai Jiao Tong University, Shanghai 200240, China.; ^3^ Key Laboratory for Laser Plasmas (MOE) and Collaborative Innovation Center of IFSA, Shanghai Jiao Tong University, Shanghai 200240, China.; ^4^ Tsung-Dao Lee Institute, Shanghai Jiao Tong University, Shanghai 201210, China.

## Abstract

The continuous development of bright x/gamma-ray sources has opened up new frontiers of science and advanced applications. Currently, there is still a lack of efficient approaches to produce high-energy gamma-rays with peak brilliance comparable to modern free-electron lasers. Here, we report a novel mechanism called beam fast pinching radiation burst to generate such gamma-ray sources. It is achieved by injecting a giga-electron volt electron beam into a submillimeter plasma with an upramp density profile, enabling violent beam pinching to occur rapidly. During this process, a burst of collimated gamma-rays is efficiently produced with photon energies ranging from mega-electron volts to giga-electron volts, electron-to-photon energy conversion efficiency above 20%, and peak brilliance exceeding 10^28^ photons s^−1^ mm^−2^ mrad^−2^ per 0.1% bandwidth. All of these are several orders of magnitude higher than existing gamma-ray sources. This opens a novel avenue for the development of extremely bright gamma-ray sources for both fundamental research and cutting-edge applications.

## Introduction

Bright x-ray sources from synchrotrons or x-ray free-electron lasers (XFELs) have become crucial tools to advance science and applications in broad areas [1], including medicine, materials science, and industry. Currently, these x-ray sources are mostly based on large-scale radio-frequency accelerators, making them available only in a few national laboratories. Moreover, the photon energy from these sources is generally limited to the range of a few kilo-electron volts (keV) to hundreds of keV. There is a huge demand and great interest in developing compact radiation sources and extending photon energy to well beyond mega-electron volts (MeV) with high efficiency and high brilliance, as they have unique applications in basic science, such as exploring strong-field quantum electrodynamics (QED) physics [2,3], probing nuclear structure and reaction dynamics [4,5], developing gamma-ray photon colliders [6], and enabling frontier research in relativistic astrophysics from gamma-ray bursts to the formation of lepton-dominated jets [7,8].

In addition to these based on the conventional accelerator technologies, plasma-based accelerators driven by either intense laser pulses or high-energy particle beams are being developed as compact accelerators and radiation sources [9–12]. A nonlinear plasma wake can support large acceleration gradients ~100 giga-volts per meter, which are more than 3 orders of magnitude higher than those found in conventional accelerators. The x-ray emission is produced via betatron radiation [13,14], nonlinear Thomson, or inverse Compton scattering [15,16]. For betatron radiation, considerable efforts have been made to enhance their photon energy and/or brilliance, for example, by the use of resonant betatron oscillations [17], a passive plasma lens [18], and density tailored plasmas [19]. They usually have limited field strength and low beam density. So far, the energy of emitted photons measured in experiments is limited to the level of a few keV to hundreds of keV, the peak brilliance is limited to the order of 10^23^ photons s^−1^ mm^−2^ mrad^−2^ per 0.1% bandwidth (BW), and the energy conversion efficiency is generally on the order of 0.001%. For nonlinear Thomson and inverse Compton scattering, the peak brilliance and the energy conversion efficiency are also relatively low and the experimental implementation is usually challenging as the requirements for spatial–temporal control between the electron beams and laser beams are very high. Nevertheless, this method of laser-electron scattering has the potential to produce gamma-rays above MeV; for example, the planned gamma-ray facility at the Extreme Light Infrastructure for Nuclear Physics (ELI-NP) [5] is expected to deliver gamma-ray pulses with photon energy up to 20 MeV and peak brilliance in the range of 10^19^ to 10^23^ photons s^−1^ mm^−2^ mrad^−2^ per 0.1% BW. Gamma-rays can also be produced by bremsstrahlung of energetic electrons [20,21] or some other proposed methods with the next generation of ultrahigh-power intense lasers [22–25]. However, these schemes usually produce gamma-rays with either relatively large divergence angles, large beam sizes, or low efficiencies, which restrict the achievable maximum photon brightness. Recent studies suggest that betatron radiation based on laser-plasma wakefield accelerators, when driven in the QED regime using multi-petawatt (PW) lasers in a 2-plasma-stage configuration, can generate collimated gamma-rays with high brightness [26]. Even so, the produced gamma-rays retain a spot size of several micrometers and a divergence over 10 mrad. Overall, despite considerable advances made so far, it is still a huge challenge to efficiently generate extremely bright gamma-ray sources with photon energies up to the giga-electron volt (GeV) level and with high peak brilliance comparable to modern XFELs. Such high-energy brilliant gamma-rays can greatly increase the photon interaction probability, thereby providing new possibilities for many cutting-edge applications, including efficient pair creation via gamma–gamma collisions for testing fundamental physics [6] and the expansion of the research scope to the GeV regime. To achieve this, not only is an efficient radiation mechanism needed to convert ultra-relativistic electrons into high-energy gamma-rays, but also the resulting beam needs to be tightly focused with high density and low divergence. How to meet these requirements simultaneously remains elusive.

In this work, we report a unique approach called the beam fast pinching radiation burst (FPRB), which can dramatically enhance the peak brilliance, photon energy, and radiation efficiency of gamma-rays emitted via direct beam–plasma interaction without relying on ultrahigh-intensity lasers. In this approach, a highly nonlinear wake is driven by an ultra-relativistic electron beam in nonuniform plasma with an upramp density profile, where the beam can be quickly pinched as a whole as it propagates in such plasma. Drastic emission of gamma-rays is produced with unprecedented high efficiency and high brightness.

## Results

### Physical scheme

**Fig. 1.**
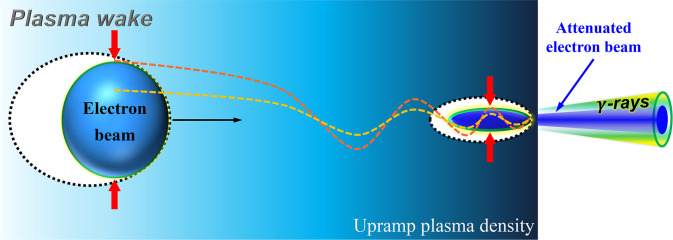
Schematic illustration. When an ultra-relativistic electron beam is incident into nonuniform plasma with an upramp density profile, the beam excites a nonlinear plasma wake. Strong beam pinching occurs due to the focusing wakefields inside the wake. The focused dense beam can, in turn, drive stronger wakefields up to tens of TV/m in a high-density plasma region, thereby leading to a burst of gamma-ray emission with extremely high efficiency and brilliance. The red arrows indicate the direction of the pinching force experienced by the electron beam. The dashed curves represent the typical trajectories of 2 electrons initially located at different transverse positions during the interaction, illustrating beam focusing and electron betatron oscillations responsible for gamma-ray emission.

Our scheme is illustrated schematically in Fig. 1. The focusing of the beam and subsequent density increase enable a nonlinear wake to be driven in high-density plasma. Note that while electron beam focusing in homogeneous plasma and the concept of plasma lenses were proposed and demonstrated before [27–29], it is difficult to focus the whole beam to a range of hundreds of nanometers and to achieve a high density above 10^22^ cm^−3^. With our approach, the beam–plasma interactions can be driven to an entirely new regime, where an unprecedented high transverse field up to tens of TV/m can be generated in the highly nonlinear wake. This field experienced by the beam electrons is so high that the QED parameter [30,31] $\chi_{e}=\gamma_{b}\left|E_{\mathrm{eff}}\right|/E_{S}$ can exceed 0.1, triggering intense emission of high-energy radiation. Here, $E_{S}=m_{e}^{2}c^{3}/\mathrm{e\hbar}\approx 1.3\times 10^{18}\;V/m$ is the QED critical field [32], $m_{e}$ is the rest electron mass, e is the elementary charge, ℏ is the reduced Planck constant, c is the speed of light in vacuum, $\gamma_{b}$ is the beam relativistic factor, $E_{\mathrm{eff}}=\left(E_{⊥}+\beta \times B\right)$ is the effective field experienced by the electron, B is the magnetic field, and $E_{⊥}$ is the electric field term perpendicular to the normalized electron velocity β=v/c. Under the combined action of the magnetic field and the electric field, the electrons undergo violent beam focusing and subsequently induce intense emission of high-energy radiation. Consequently, collimated high-energy gamma-rays are effectively emitted with an efficiency several orders of magnitude higher than existing sources, and in particular, the resulting gamma-ray brilliance can be increased to near levels of modern XFELs and the photon energy can be extended into the GeV range. Such powerful gamma-rays could provide new capabilities for fundamental research and various applications.

**Fig. 2.**
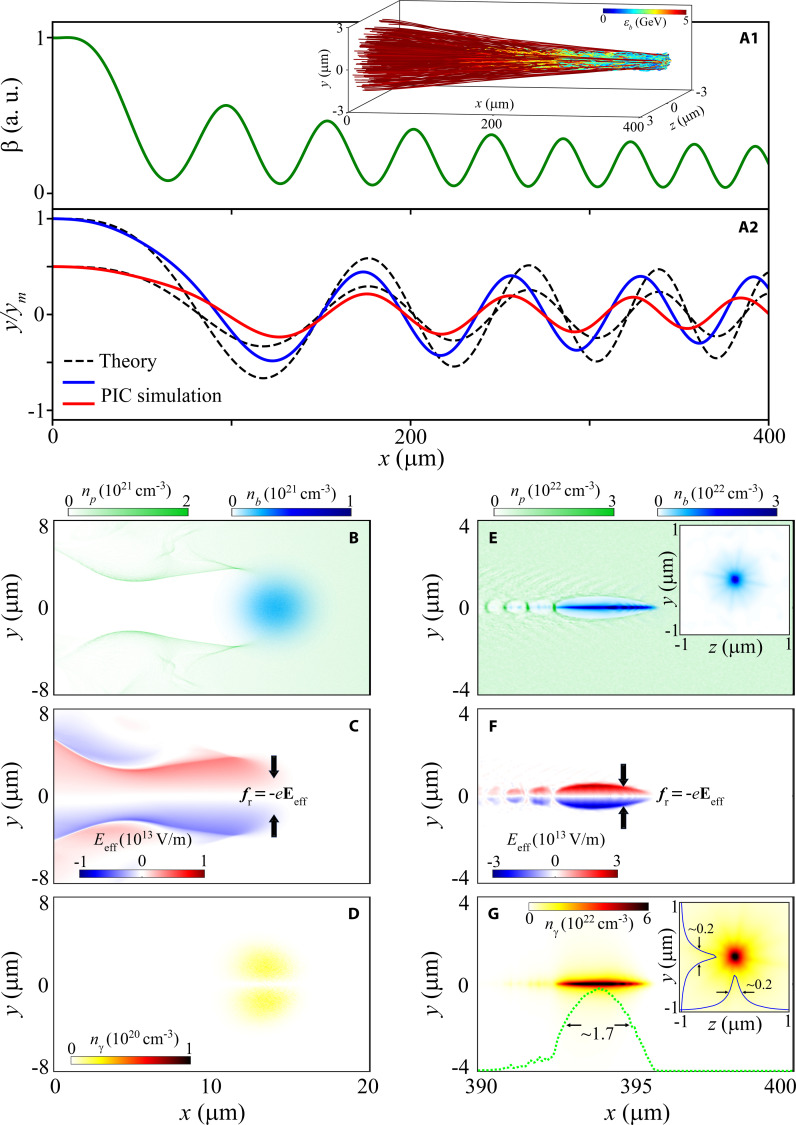
The evolution of β is shown in (A1), where the inset illustrates the evolution of the beam focusing and corresponding energy during the interaction obtained from PIC simulation. In (A2), the solid curves illustrate the typical trajectory of beam electrons obtained from PIC simulation, where the dashed curves represent the particle trajectory derived from the theory model. Distributions of (B and E) the plasma density ($n_{p}$) and the electron beam density ($n_{b}$), (C and F) the effective field ($E_{\mathrm{eff}}$), and (D and G) the photon beam density ($n_{\gamma }$) are shown at the beginning x=20μm (left column: B to D) of the plasma target and the target end x=400μm (right column: E to G), respectively. The inset in (E) exhibits the transverse distribution of the final electron beam. The inset in (G) displays the transverse distribution of the final gamma-ray beam.

We begin by discussing the model of beam focusing in nonuniform plasma. It is well known that when a relativistic electron beam passes through underdense plasma, a nonlinear wake can be excited with a bubble-like cavity around the beam, where the plasma electrons are expelled away, leaving a column of ions behind. If a beam driver propagating through the plasma can be focused along its path, its density can be enhanced correspondingly. Subsequently, if nonuniform plasma with an upramp density profile is taken to match the variation of the beam density, then this beam can excite a highly nonlinear wake even in dense plasma. This in turn leads to even more violent beam-focusing than that found in uniform plasma. Electron beam transport and focusing in longitudinally nonuniform plasma were studied before [33,34], where attention had been paid mostly on the beam emittance control. In the beam-driven nonlinear wake with the cylindrical symmetry, the focusing force felt by an electron in the beam can be described as [35] $f_{⊥}=-m_{e}\omega_{p}^{2}r/2$, which is proportional to the radial distance r. If neglecting the change of the beam particle energy during their transport, the motion of a beam particle in the transverse directions in the plasma wake is decoupled and can be described as

$$y^{\prime \prime }\left(x\right)+K\left(x\right)y\left(x\right)=0,$$ (1)

for a given transverse direction y, where x is the coordinate along the beam propagation direction, $K\left(x\right)=\frac{\omega_{p}^{2}\left(x\right)}{2\gamma_{b}c^{2}}$ is the focusing strength as a function of x through the particle energy and the local plasma density profile, $\gamma_{b}$ is the relativistic factor, and the plasma frequency is defined as $\omega_{p}\left(x\right)=\sqrt{4\pi e^{2}n_{p}\left(x\right)/m_{e}}$. The length scale of the motion can be described by the betatron wave number defined as $k_{\beta }\left(x\right)=\sqrt{K\left(x\right)}$. When the plasma density changes slowly with x, that is, $\frac{2\pi }{\;k_{\beta }^{2}\left(x\right)}\left|\;k_{\beta }^{\prime }\left(x\right)\right|\ll 1$, one can obtain an approximation solution using the Wentzel–Kramers–Brillouin (WKB) method. The particle motion can be described by the transport matrix *M* based on its initial conditions [33,34]

yy′=M11M21M12M22y0y0′, (2)

where the elements of the matrix are $M_{11}\;=\;\sqrt{\frac{\beta_{m}}{\beta_{m0}}}\left(\cos\phi +\alpha_{m0}\sin\phi \right)$, $M_{12}=\sqrt{\beta_{m}\beta_{m0}}\sin\phi$, $M_{21}=\frac{\left(\alpha_{m0}-\alpha_{m}\right)\cos\phi -\left(1+\alpha_{m0}\alpha_{m}\right)\sin\phi }{\sqrt{\beta_{m}\beta_{m0}}}$, and $M_{22}=\sqrt{\frac{\beta_{m0}}{\beta_{m}}}\left(\cos\phi -\alpha_{m0}\sin\phi \right)$, respectively, the subscript 0 indicates the initial value of a variable, $\beta_{m}=1/k_{\beta }$, $\alpha_{m}=-\frac{1}{2}\frac{d\beta_{m}}{\mathrm{dx}}$, and $\phi =\int_{0}^{x}k_{\beta }\left(s\right)\mathrm{ds}$. The spot size of the beam can be defined as the root-mean-square deviation of particle positions from the beam centroid $\sigma_{y}=\sqrt{\langle y^{2}\rangle +{\langle y\rangle }^{2}}$, where $\langle \cdot \rangle$ denotes the ensemble average, y is the transverse position of a single particle, and $\langle y\rangle$ is the beam centroid position (assumed to be 0 for centered focusing, i.e., $\langle y\rangle =0$), simplifying the beam spot size to $\sigma_{y}=\sqrt{\langle y^{2}\rangle }$. The geometric emittance can be defined as $\epsilon =\sqrt{\langle y^{2}\rangle \langle y^{\prime 2}\rangle -{\langle yy^{\prime }\rangle }^{2}}$. Then, it can be deduced that

$$\langle y^{2}\rangle =\epsilon_{0}\beta_{m}\left(A+B_{1}C+B_{2}S\right),$$ (3)

where $\epsilon_{0}=\sqrt{\langle y_{0}^{2}\rangle \;\langle y_{0}^{\prime 2}\rangle -{\langle y_{0}y_{0}^{\prime }\rangle }^{2}}$, $A=\frac{1}{2}\left(\beta_{0}\gamma_{m0}+\gamma_{0}\beta_{m0}-2\alpha_{0}\alpha_{m0}\right)$, $B_{1}=\frac{\beta_{0}}{\beta_{m0}}-A$, $B_{2}=\alpha_{m0}\frac{\beta_{0}}{\beta_{m0}}-\alpha_{0}$, $C=\int \mathrm{d\phi}f_{\phi }\left(\phi \right)\cos\left(2\phi \right)$, S= $\int \mathrm{d\phi}f_{\phi }\left(\phi \right)\sin\left(2\phi \right)$, $\beta_{0}=\langle y_{0}^{2}\rangle /\epsilon_{0}$, $\gamma_{0}=\langle y_{0}^{\prime 2}\rangle /\epsilon_{0}$, $\alpha_{0}=-\langle y_{0}y_{0}^{\prime }\rangle /$ $\epsilon_{0}$, $\gamma_{m}=\left(1+\alpha_{m}^{2}\right)/\beta_{m}$, and $f_{\phi }\left(\phi \right)$ is the distribution function of the betatron phase advance. Assuming that the beam does not have energy spread, the relationship between the geometric emittance and the spot size can be defined as $\sigma_{y}^{2}=\epsilon \beta \left(x\right)$. Then, one can approximately describe the model to include the evolution of the beam size via the differential equation for the beta function [33,34]

$$\frac{1}{2}\beta \left(x\right)\beta^{\prime \prime }\left(x\right)-\frac{1}{4}\beta^{\prime }{\left(x\right)}^{2}+\beta {\left(x\right)}^{2}k_{\beta }^{2}\left(x\right)=1,$$ (4)

which can be solved numerically alongside the single-particle motion. Numerical calculation of Eqs. (1) and (4) shows that a proper density upramp is key for achieving strong beam focusing in plasma, as shown in Fig. 2A1 and A2 for the beam and plasma conditions given in the following.

The rapid pinching of the electron beam in plasma leads to dramatic increase in the beam density. As a result, nonlinear wakes with unprecedented high fields up to tens of TV/m can be produced in the high plasma density region, enabling a new kind of beam-driven gamma-ray radiation. Under this condition, the electrons undergo giant transverse forces, which can greatly enhance the quantum parameter $\chi_{e}=\gamma_{b}\left|f_{⊥}\right|/eE_{S}$ and thus naturally lead to the emission of high-energy photons [36], where $\left|f_{⊥}\right|\approx 2\pi e^{2}n_{p}r$. The photon energy $\varepsilon_{\gamma }$ radiated by an electron in strong external fields can be derived from the probability spectrum distribution $\begin{array}{c} \begin{array}{c} \begin{array}{c} \mathrm{dW}\approx \frac{m_{e}^{2}c^{4}\alpha_{f}}{\sqrt{3}\pi \hbar \varepsilon_{e}}\frac{\mathrm{du}}{{\left(1+u\right)}^{3}}\left\{\left[1+{\left(1+u\right)}^{2}\right]K_{2/3}\left(\frac{2u}{3\chi_{e}}\right)-\left(1+u\right)\int_{2u/3\chi_{e}}^{\infty }\mathrm{dy}K_{1/3}\left(y\right)\;\right\} \end{array} \end{array} \end{array}$, which is suitable for arbitrary field configurations if the local-constant-field approximation holds [32]. Here, $\alpha_{f}=e^{2}/\hbar c$ is the fine-structure constant, $K_{2/3}$ and $K_{1/3}$ are the modified Bessel function of the second kind, $u=\varepsilon_{\gamma }/\left(\varepsilon_{e}-\varepsilon_{\gamma }\right)$, and $\varepsilon_{e}$ is the initial electron energy. Since the energy of most radiated photons is much less than that of electrons, the characteristic photon energy can be conveniently approximated as $\langle \varepsilon_{\gamma }\rangle \approx \frac{4}{5\sqrt{3}}\varepsilon_{e}\chi_{e}$.

### High-efficiency gamma-ray generation

It should be noted that the above analysis model can only roughly describe the dynamics of beam focusing. This is because strong radiation effects lead to substantial changes in beam energy, and there are also highly nonlinear effects. In order to demonstrate the proposed scheme and illustrate the beam pinching and radiation processes quantitatively, 3-dimensional (3D) particle-in-cell (PIC) simulations were performed (see Methods for more details on the configuration and simulation setup). As an example, we take an electron beam driver, which has about 2 nC charge, 5 GeV mean energy, 5 mm-mrad normalized emittance, an energy spread of 5% full width at half maximum (FWHM), and a Gaussian density distribution of $n_{b}=n_{b0}\exp\left(-\frac{r^{2}}{2\sigma_{r0}^{2}}-\frac{{\left(x-\mathrm{vt}-x_{1}\right)}^{2}}{2\sigma_{x0}^{2}}\right)$, where $\sqrt{2}\sigma_{x0}=2\;\mu m$, $x_{1}=-3\sqrt{2}\sigma_{x0}$, $\sqrt{2}\sigma_{r0}=2.5\;\mathrm{\mu m}$, and $n_{b0}=2\times 10^{20}\;{\mathrm{cm}}^{-3}$. A nonuniform plasma slab with an upramp density is used as a converter to realize fast pinching of the electron beam and subsequent efficient emission of high-energy photons. The plasma target has a linearly increasing density profile $n_{p}=n_{p0}\left(x/L\right)$ ranging from 0 to $n_{p0}=5\times 10^{21}\;{\mathrm{cm}}^{-3}$ over the longitudinal distance of L=400μm. More information on the parameters of the electron beam and plasma target can be found in Methods.

Figure 2 shows the simulation results for strong beam focusing and dense gamma-ray emission. During the beam–plasma interaction, the electrons undergo intense oscillations while the beam as a whole undergoes strong focusing, as illustrated in Fig. 2A. Here, the inset reflects the collective behavior of the entire beam electron ensemble, demonstrating the characteristics of beam focusing and radiation energy loss. Figure 2A1 and A2 illustrate the evolution of β and the selected trajectories of beam particles according to Eqs. (4) and (1), respectively. These trajectories exhibit distinct oscillation characteristics. Note that there are differences in the electron trajectories shown in Fig. 2A2 between the theoretical model and PIC simulation results, which is partially due to the neglecting of the particle energy loss via radiation and other strong nonlinear effects in the theory model. As it shows, the beam radius decreases along the propagation distance quickly, so its density increases significantly according to $n_{b}\propto n_{b0}\sigma_{r0}^{2}/r^{2}$ (Fig. 2B compared with Fig. 2E). The results show that the beam pinching and radiation effects can be substantially enhanced by the proposed interaction configuration. The focused beam passing through the plasma upramp can in turn excite extremely strong transverse fields of more than $10^{13}\;V/m$ (see Fig. 2F), further enhancing both beam pinching and photon emission. During the interaction, the QED parameter $\chi_{e}$ can exceed 0.2, allowing a large amount of beam energy to be effectively converted into intense gamma-ray emission. Hence, each radiated photon can gain a large fraction of the electron energy, with a cutoff energy of more than 1 GeV. Because the focused electron beam has an ultra-high density and submicrometer diameter, it has the ability to produce an ultra-intense gamma-ray beam of similar small size and high density (see Fig. 2G), resulting in unprecedented high brilliance, several orders of magnitude higher than current gamma-ray sources.

**Fig. 3.**
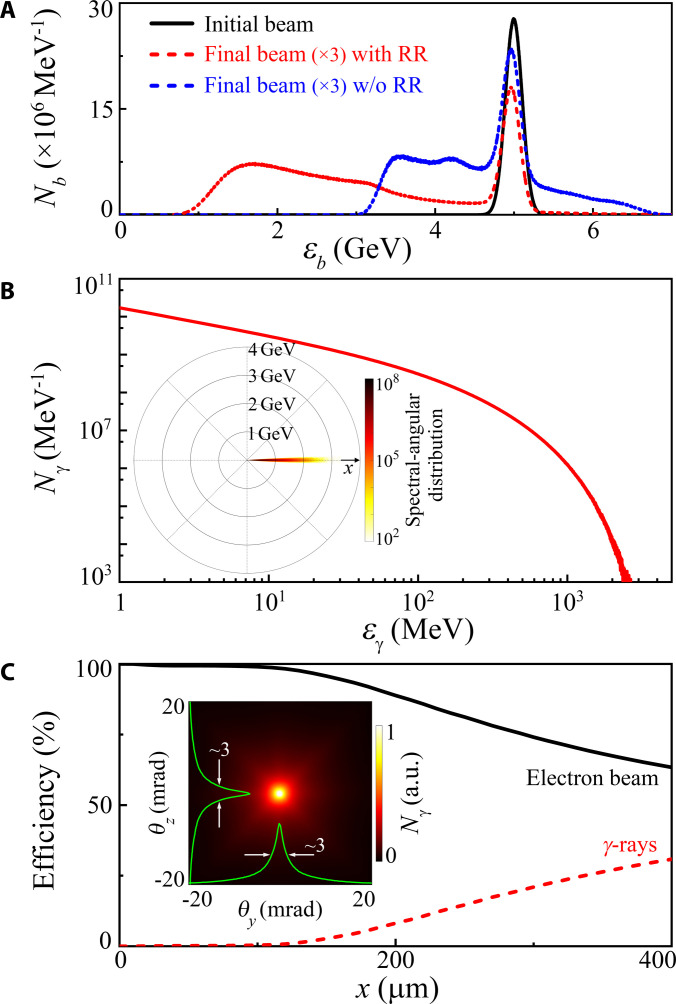
(A) The initial (black line) and final (red line) energy spectrum of the electron beam, where the blue line depicts the final beam energy spectrum without radiation reaction effect. (B) The final energy spectrum of emitted gamma-rays, where the inset shows the spectral angular distribution as a function of photon energy. (C) Evolution of the energy efficiency of the electron beam (black line) and gamma-rays (red line), where the inset shows the angular distribution of gamma-rays.

In order to save computational resources, we adopt a beam driver with a range of $\sqrt{2}\sigma$ in each direction in the following parameter study. This means that the total charge of the beam will be reduced to approximately 1 nC. When all of the beam charge is loaded, the edges of the beam may not be able to be fully focused, but the underlying mechanisms and relevant physical effects do not change. The simulation results show that the radiation efficiency using a beam with a larger range of $3\sqrt{2}\sigma$ is approximately 65% of that using the beam with the range of $\sqrt{2}\sigma$. Figure 3 shows the energy spectrum of the electron beam and gamma-rays and their energy efficiency evolution. Because of the conversion of a large amount of electron beam energy into intense gamma-ray emissions, the electron energy spectrum is radically changed from a single peak of 5 GeV to a broad spectrum with 2 peaks of approximately 1.6 and 5 GeV. After the interaction with the plasma in a length of 400 μm, the energy conversion efficiency of the electron beam to gamma-rays is as high as nearly 31%, producing photons with high energies ranging from MeV to GeV. Most of the gamma-ray photons are generated near the axis, with an average energy of approximately 70 MeV. The gamma-ray source has a small divergence of approximately 3 mrad FWHM. According to the spatial distribution of the generated gamma-rays shown in Fig. 2G and the energy spectrum shown in Fig. 3B, one can obtain the source size of about 0.2μm, the duration of about 5 fs, and $1.8\times 10^{10}$ photons at 1 MeV. These correspond to a peak brilliance of about $1\times 10^{28}$ photons s^−1^ mm^−2^ mrad^−2^ per 0.1% BW. This is about 5 orders of magnitude higher than existing sources. Note that as the gamma-rays are produced simultaneously during the electron beam pinching process, they co-propagate with the beam. Hence, although radiation emission persists throughout the entire pinching process, the resulting gamma-ray pulse does not stretch temporally; instead, it has a duration comparable to the focused beam duration.

To examine the role of radiation reaction (RR) effect in the scheme, we performed an additional simulation by turning off the RR effect. As can be seen from Fig. 3A, there are obvious differences in electron beam energy spectra with and without the RR effect. In the absence of the RR effect, there is a distinct high-energy part of the spectral distribution above 5.3 GeV. This is mainly because the electrons at the tail of the beam are accelerated in the wake acceleration phase. Meanwhile, because of the wakefield excitation and the deceleration of some beam electrons at the beam head, the energy spectrum appears a low-energy tail distributed below 4.7 GeV. In contrast, in the case with the RR effect included, there is no high-energy part of the beam energy spectrum beyond 5.3 GeV due to the substantial energy loss via radiation emission, where its low-energy tail is more distinct, extending to 0.8 GeV. These clearly demonstrate that the RR effect plays a crucial role in the electron beam energy evolution, even if its stochastic and discrete nature cannot be directly observed from the single electron trajectory. As a result, as high as 31% of the beam energy can be converted into intense gamma-ray emission. Therefore, in order to reveal the intrinsic physics of strong beam–plasma interactions, the radiation effect must be taken into account.

As a comparison, we also studied the beam propagation in uniform plasma to demonstrate the importance of the plasma ramp configuration for efficient beam focusing and gamma-ray emission (see the Supplementary Materials for more details). Because of the lack of effective beam focusing in uniform plasma, only about 0.3% of the beam energy can be converted into gamma-rays with a large transverse size. The corresponding gamma-ray brilliance is approximately 4 orders of magnitude lower than that produced in the upramp nonuniform plasma.

**Fig. 4.**
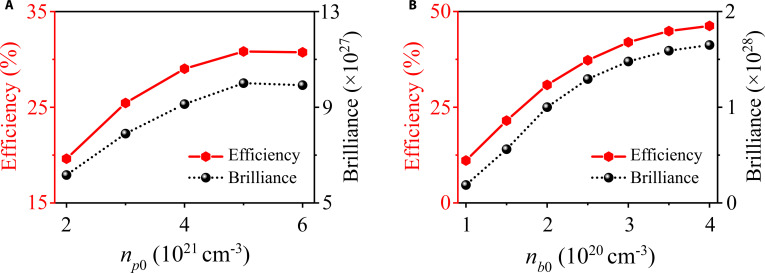
Effects of (A) the plasma density ($n_{p0}$), and (B) beam density ($n_{b0}$) on the gamma-ray energy efficiency and peak brilliance (photons s^−1^ mm^−2^ mrad^−2^ per 0.1% bandwidth).

We now demonstrate the robustness of this scheme in terms of the beam and plasma parameters. We first investigate the effect of the maximum density of the plasma ramp (equivalent to density gradient shift) on photon emission, while keeping other parameters fixed. The results show that a proper high density is beneficial to beam focusing and field enhancement in plasma, leading to more efficient photon emission and higher brilliance, as shown in Fig. 4A. Nevertheless, the plasma ramp density cannot be arbitrarily high; Otherwise, the beam driver cannot be well focused and confined in its excited wake, resulting in attenuation of radiation emission.

Figure 4B illustrates the effect of the initial beam density on the gamma-ray radiation. The corresponding change in electron beam charge is determined by $Q_{b0}\propto n_{b0}$ for a given beam size. In order for the similarity ratio parameter $R=n_{b0}/n_{p0}$ to be constant, the plasma density varies with the initial beam density, while all other parameters remain unchanged. As expected, the beam with a higher initial density can dramatically boost the photon emission because it can drive more denser plasma, thereby exciting stronger focusing fields and thus generating larger parameter $\chi_{e}$. Therefore, the radiation efficiency and peak brilliance of emitted gamma-rays can be dramatically enhanced. For example, when $n_{b0}=4\times 10^{20}{\mathrm{cm}}^{-3}$, the gamma-rays have an energy efficiency of up to 46.3% and a high brilliance of about $1.7\times 10^{28}$ photons s^−1^ mm^−2^ mrad^−2^ per 0.1% BW. It is worth mentioning that, as a large amount of the electron beam energy is lost, the growth in radiation efficiency and peak brilliance gradually slows down both with the further increase of plasma density and beam density.

**Fig. 5.**
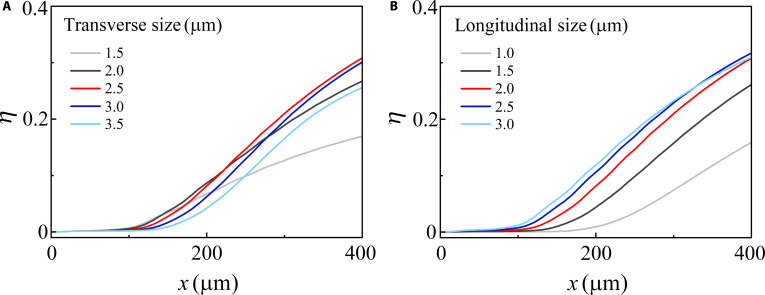
Energy conversion efficiency (η) of gamma-rays emitted as a function of the interaction distance for (A) different transverse beam sizes with fixed $\sqrt{2}\sigma_{x0}=2\;\mathrm{\mu m}$, and (B) different longitudinal beam sizes with fixed $\sqrt{2}\sigma_{r0}=2.5\;\mathrm{\mu m}$. Here, η is defined as the ratio of the gamma-ray pulse energy to the initial electron beam energy.

We also investigate the effects of the beam driver sizes on photon emission in Fig. 5. It is shown that an appropriately large beam is beneficial for efficient generation of bright gamma-rays. If the electron beam has a small radius or a short length (corresponding to a lower beam charge according to $Q_{b0}\propto n_{b0}\sigma_{r0}^{2}\sigma_{x0}$), it means that the final beam density after focusing will decrease, so that the beam may not be able to excite a strong enough focusing field. This may cause a decrease in the radiation efficiency of gamma-rays. For example, when using a driving electron beam with $\sqrt{2}\sigma_{r0}=1.5\;\mathrm{\mu m}$ and about 400 pC charge, the radiation efficiency drops to approximately 17%, but it is still several orders of magnitude higher than that of existing wakefield radiation mechanisms. On the other hand, the beam size should not be too large; otherwise, it will not be fully confined within the plasma wake, leading to a decrease in the energy efficiency of radiation. Overall, the scheme can be further optimized by changing the size of the electron beam, its density, and the plasma density profile, etc., thus further improving the beam focusing and the resulting photon emission.

To further investigate the effects of the beam and plasma parameters, we have performed additional simulations to keep the beam charge constant when varying the bunch length and spot size, as well as to change the plasma ramp length, as detailed in the Supplementary Materials. Additional simulations indicate that in plasma with a longer density scale length, it is beneficial to convert more electron energy into gamma-ray emission, thereby achieving a high radiation efficiency of over 40%. All these suggest that our scheme is robust and effective for a broad range of beam and plasma parameters.

## Discussion

**Fig. 6.**
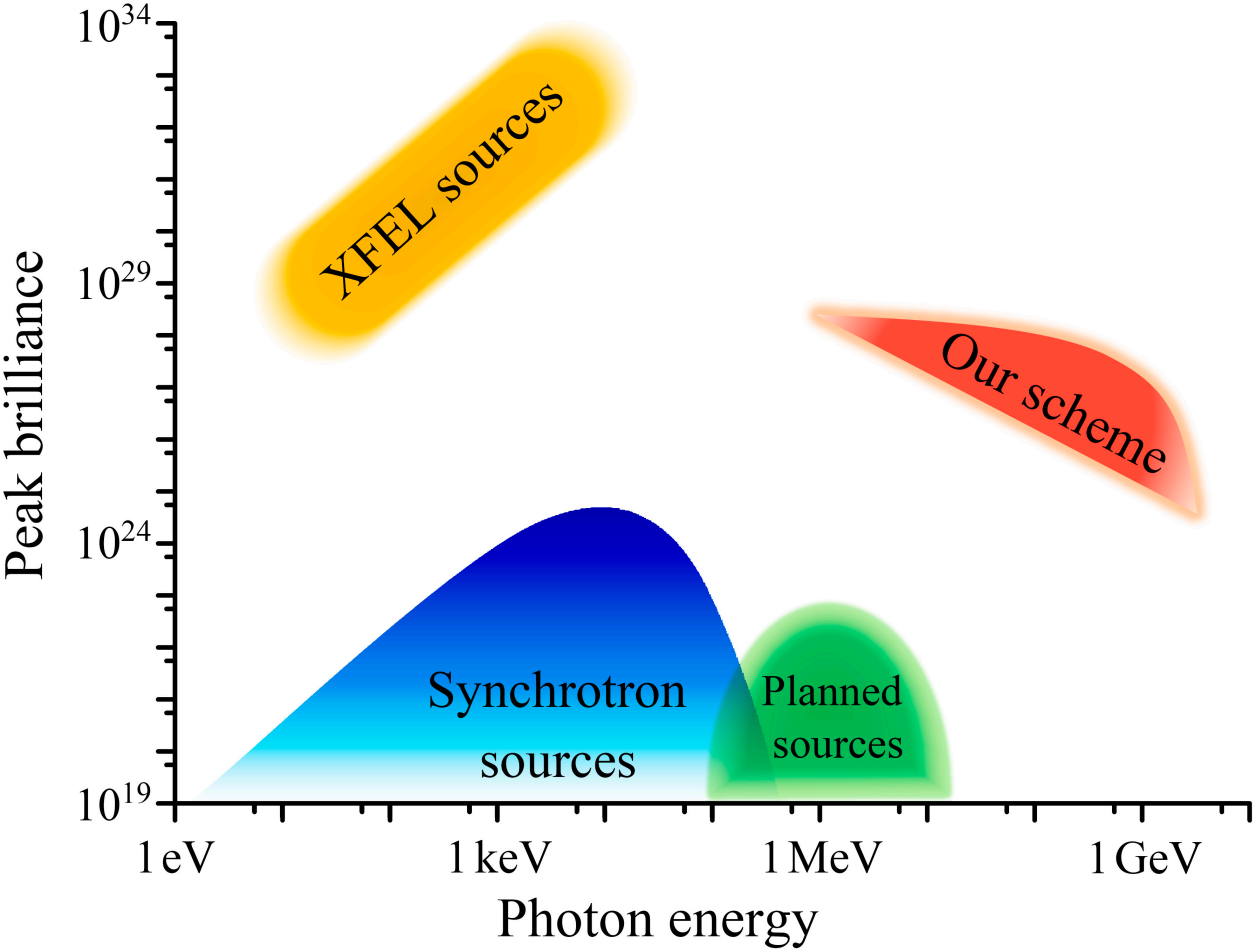
Comparison of our proposed source with other sources in terms of the peak brilliance (photons s^−1^ mm^−2^ mrad^−2^ per 0.1% bandwidth). The plots show schematically summarized properties of XFEL sources (yellow), synchrotron sources (blue), and the planned gamma-ray sources (green) at the ELI-NP. The red area indicates the prospects of our proposed source, which is estimated based on the results of 3D PIC simulations.

We now discuss the capability comparison between our proposed source and other existing sources. As illustrated in Fig. 6, the proposed source exhibits distinctive advantages—it can achieve ultrahigh peak brilliance within an energy range inaccessible to conventional sources. Moreover, the gamma-ray beams produced by our source have an extremely small spot size on the order of 0.1 μm and with a high density exceeding 10^22^ cm^−3^. Such exceptionally high brilliance and dense photon distribution will significantly enhance the probability of collisions. This source, characterized by a high flux and a wide spectrum with photon energies reaching the GeV level, would be a powerful tool for investigating fundamental QED physics [2,3,6] and is expected to open up new possibilities for exploring photonuclear reactions [4,5] and extreme astrophysical phenomena [7,8].

In summary, we have discovered the FPRB mechanism for efficient and ultrabright gamma-ray emission as a relativistic electron beam propagates in plasma with an upramp density profile. The beam is found to be focused dramatically as a whole to a submicrometer diameter via a beam pinching process, increasing its density by hundreds of times. Such a dense beam in turn drives the plasma wake to unprecedented fields of tens of TV/m, enabling one to access the radiation-dominated regime. Under such extreme conditions of interaction, more than 20% of the beam energy can be converted into intense gamma-ray emission, where the photon energy can be extended to the GeV level. Compared with previously proposed methods based on electron–solid and laser–electron collisions [37–39], our scheme shows a few merits, such as high efficiency and high brilliance in the produced gamma-rays, and can be realized in a relatively simple configuration. As an example, the resulting gamma-ray brilliance can reach an unprecedented level above 10^28^ photons s^−1^ mm^−2^ mrad^−2^ per 0.1% BW, which is about 5 orders of magnitude higher than existing sources. This opens an efficient and promising route for the development of compact bright gamma-ray sources, which is of great importance and interest for both fundamental research and applications [2–8].

## Methods

To demonstrate the proposed scheme, 3D PIC simulations were carried out using the QED-PIC code EPOCH [40]. In the simulation, a density gradient plasma is adopted, which has a linearly increasing density profile ranging from 0 to $5\times 10^{27}m^{-3}$ over the longitudinal distance of 400μm. Note that this linear density profile is adopted as an example for simplicity, and our scheme also works for other plasma profiles with positive density gradients (e.g., a quadratically increasing density profile, a long density gradient, and a stair-like or layered density profile), as discussed in the Supplementary Materials. There are several methods to generate this type of plasma structures in experiments, such as the prepulse laser heating [41] and supersonic gas jets with a blade [42]. For the simulation results in Fig. 2, we take an example of an electron beam with velocity v moving along the *x*-axis, which is initialized at a waist in vacuum. The beam driver has about 2 nC charge, 5 GeV mean energy, 5 mm-mrad normalized emittance, an energy spread of 5% FWHM, and a Gaussian density distribution with $\sqrt{2}\sigma_{x0}=2\;\mathrm{\mu m}$, $x_{1}=-3\sqrt{2}\sigma_{x0}$, $\sqrt{2}\sigma_{r0}=2.5\;\mathrm{\mu m}$, and $n_{b0}=2\times 10^{20}{\mathrm{cm}}^{-3}$. Although these parameter settings are somewhat too high for most accelerator laboratories, comparable beam parameters are achievable with advanced techniques, such as the laser-plasma accelerators or advanced RF accelerators in combination with a plasma lens [43]. For instance, some of the experimental plans at FACET-II (e.g., E-332, E-308, and E-335) [44] are explicitly designed to achieve such GeV, nC, and μm-scale electron beams with parameters comparable to or even superior than those adopted in our scheme. It should be noted that, more recently, 10-GeV, ~0.1-MA ultrahigh current femtosecond-scale electron beams with PW peak power in particle accelerators have been realized [45]. Another alternative is the laser wakefield accelerator. According to the scaling laws for the beam charge $Q\propto \sqrt{P}$ and energy gain $∆E\left[\mathrm{GeV}\right]\propto P^{1/3}$ [46], it can be predicted that when using PW-class laser pulses, an electron beam with an energy of multi-GeV and a charge reaching up to the nC level can be obtained. For example, in recent experiments, a PW-class laser was utilized to generate such high-charge high-energy electron beams with energy up to 10 GeV and about 1.7 nC charge [47]. Such PW laser systems are commercially available and established in laboratories worldwide. These clearly validate the feasibility of the electron beam parameters adopted in our work. For the parameters considered, the beam self-current is more than 3 orders of magnitude lower than the Alfvén current limit $I_{A}=m_{e}\gamma_{b}c^{3}/e\approx 17\gamma_{b}\;\mathrm{kA}$ . A simulation window moving along the *x* direction with velocity c is employed, which has a size of $20\;\mathrm{\mu m}\;\left(x\right)\times 20\;\mathrm{\mu m}\;\left(y\right)\times 20\;\mathrm{\mu m}\;\left(z\right)$ with 1,000×1,000×1,000 grid cells. The numbers of macro-particles in each cell for the beam electrons and plasma electrons (ions) are 27 and 8 (8), respectively.

Both beam and target parameters are tunable, as shown in Figs. 4 and 5. The lowest beam charge among them is approximately 400 pC, which can be produced with 100-TW-class laser pulses. Our scheme also works for electron beams with a relatively large energy spread as usually found in laser wakefield accelerators. In additional simulations, by adopting an electron beam with 10% energy spread, it is found that our scheme continues to work well. Physically, as considerable energy of electrons is transferred into intense gamma-ray radiation during the beam–plasma interaction, it will naturally lead to a substantial broadening of the electron beam energy spectrum. Such inherent energy loss and spectral broadening effects greatly relax the constraint on electron beam monochromaticity. Furthermore, our scheme also works well for electron beams with smaller charges such as those at the hundreds of pC level, which further lowers the experimental requirements.

## Data Availability

The data that support the plots and findings of this study are available from the corresponding authors upon reasonable request.
