# Supplementary material for: High-Efficiency Radiation via Fast Electron Beam Pinching in Nonuniform Plasmas
Source: Research (Wash D C). 2026 Jun 19;9:1330. doi: 10.34133/research.1330 (PMC13280933; doi:10.34133/research.1330)
Supplement: Supplementary 1 — Figs. S1 to S6 [file research.1330.f1.pdf]

Supplementary Materials for

High-Efficiency Radiation via Fast Electron Beam Pinching in Nonuniform Plasmas

Xing-Long Zhu[1], Min Chen[2, 3], and Zheng-Ming Sheng[2, 3, 4]

[1] Institute for Fusion Theory and Simulation, School of Physics, Zhejiang University, Hangzhou 310058, China

[2] State Key Laboratory of Dark Matter Physics, School of Physics and Astronomy, Shanghai Jiao Tong University, Shanghai 200240, China

[3] Key Laboratory for Laser Plasmas (MOE) and Collaborative Innovation Center of IFSA, Shanghai Jiao Tong University, Shanghai 200240, China

[4] Tsung-Dao Lee Institute, Shanghai Jiao Tong University, Shanghai 201210, China

1. Interaction of relativistic electron beams with homogeneous plasma

As a comparison, we investigate the interaction of an ultra-relativestic electron beam with an uniform plasma, as shown in Fig. S1. The density of the homogeneous plasma is set to half of the peak density of the inhomogeneous plasma with a linearly increasing density profile to have a good comparison for the two cases. Meanwhile, other parameters remain the same as those in the case of the positive density gradient plasma. In the uniform density plasma, the initial driving electron beam density ($n_b \ll n_p$) is too low to excite a nonlinear plasma wake, making it difficult for the beam to be focused. It should be mentioned that strong self-generated electromagnetic fields can be induced during the interaction due to the development of the filamentation instability. However, the excited field strength is about an order of magnitude lower than that produced in the case of the density gradient plasma. This means that the QED parameter χ_e will also be reduced by about an order of magnitude to much less than 0.1. As a result, only about 0.3% of the electron beam energy can be converted into gamma-rays, and the photon energy and yield of the emitted gamma-rays will be significantly reduced. In addition, the resulting gamma-ray source has a considerable large size, which is comparable to that of the initial beam driver. Due to the lack of effective beam focusing and intense radiation emission, the brilliance

of the emitted gamma-ray source is approximately four orders of magnitude lower than that produced in the nonuniform plasma with an upramp density gradient.

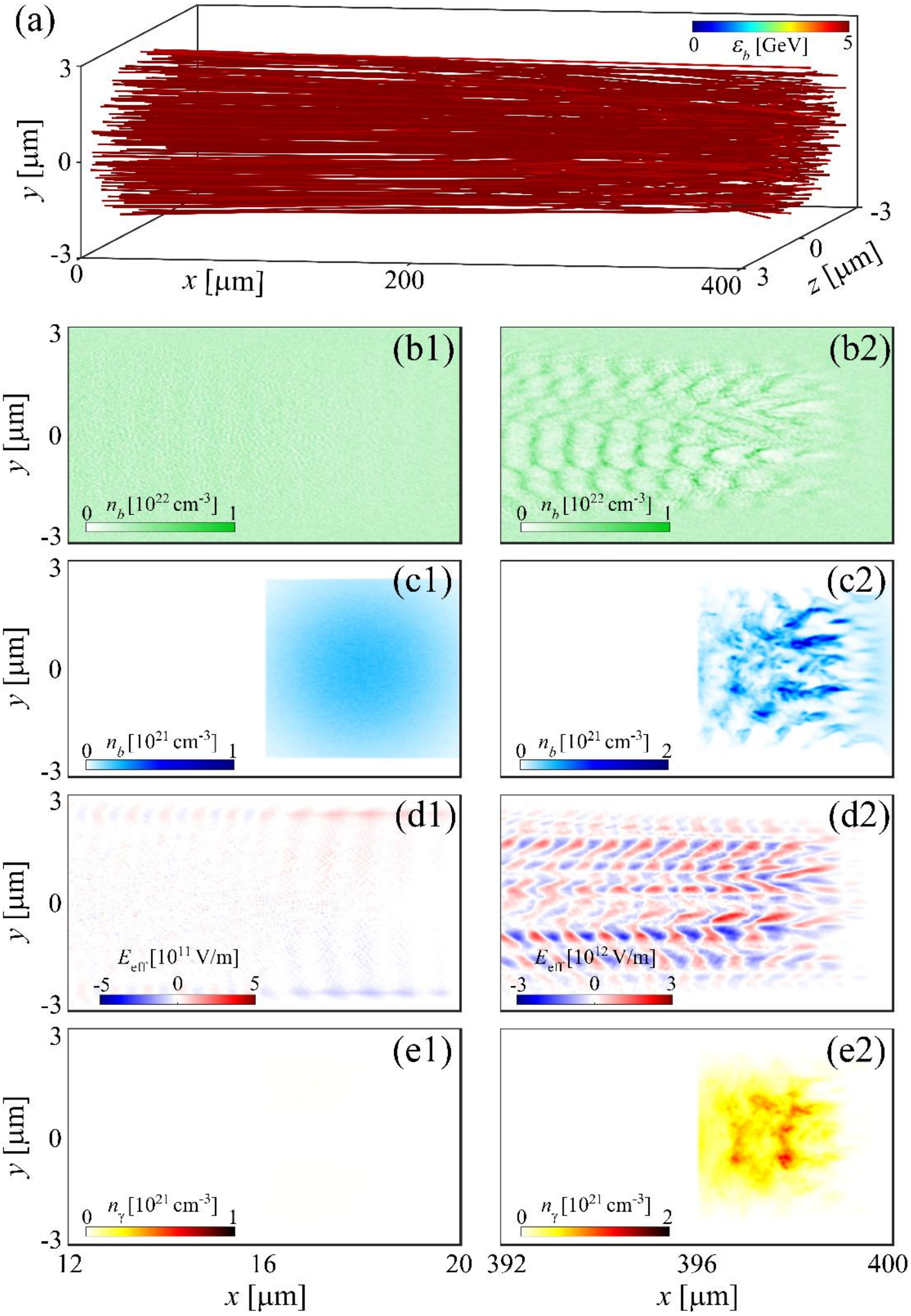

Fig. S1 | The case for beam propagation in homogeneous plasma. (**a**) Evolution of the energy trajectory of selected beam electrons. Distributions of (**b1** and **b2**) the plasma density (n_p), (**c1** and **c2**) the beam density (n_b), (**d1** and **d2**) the effective interaction field ($E_{\rm eff}$) and (**e1** and **e2**) the photon density (n_γ) are shown at the beginning (left column) of the plasma target and the target end (right column), respectively.

2. Effects of nonuniform plasma with different density profiles

To demonstrate the robustness of the scheme, we investigate the effect of the plasma density profile in Fig.

S2. Here, we adopt two commonly used plasma profiles with a linear increasing density distribution given by the function $n_p = n_{p0}(x/L)$ and a quadratically increasing density distribution given by the function $n_p = n_{p0}(x^2/L^2)$, respectively, while all other parameters are kept the same. In addition, to further investigate the potential of this scheme, the electron beam continues to interact with a 200μm homogeneous plasma with $n_p = n_{p0}$ after passing through a density gradient plasma with a length of $L = 400$μm. It is shown that the beam focusing and induced photon emission occur earlier in the case of $n_p = n_{p0}(x/L)$, and the resulting gamma radiation efficiency is higher than that in the case of $n_p = n_{p0}(x^2/L^2)$. This is mainly because when n_{p0} is fixed, the electron beam will experience a larger excitation field in plasma with a density profile $n_p = n_{p0}(x/L)$ over the same distance, compared to the case of $n_p = n_{p0}(x^2/L^2)$, resulting in stronger beam focusing and subsequent stronger radiation. In both cases, the electron beam undergoes significant beam pinching and excites large plasma fields. Once strong beam focusing occurs, it quickly triggers intense emission of high-energy photons, producing giant gamma-rays with high efficiency. In addition to the energy conversion efficiency, other characteristics of the gamma-rays produced in both cases are very similar.

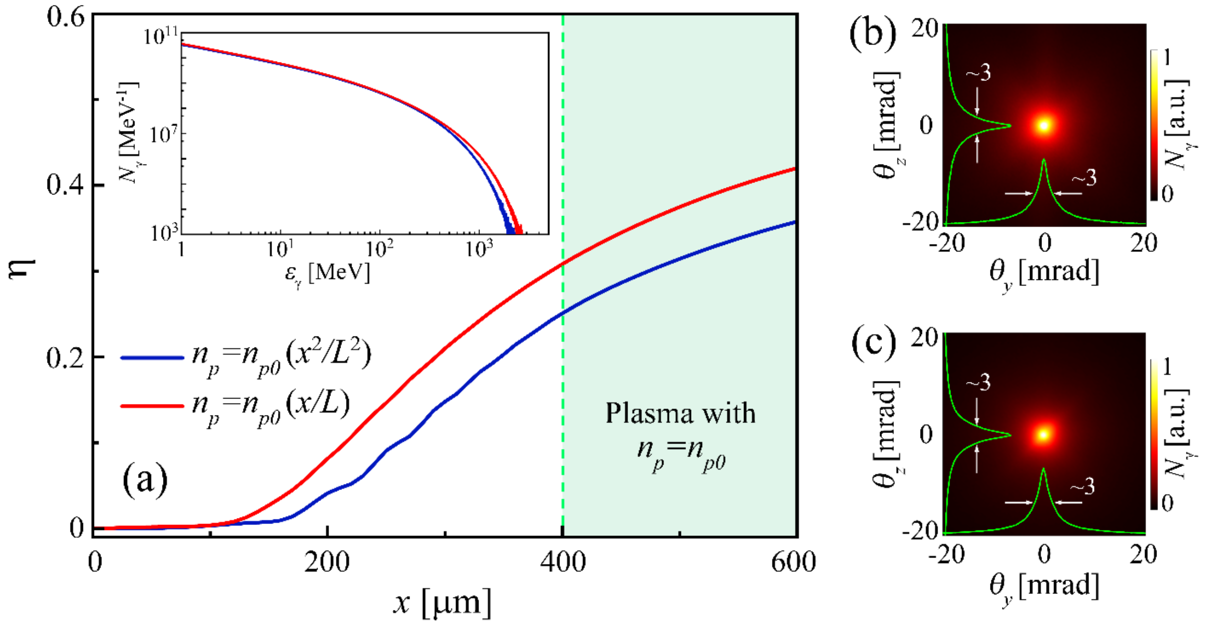

Fig. S2 | Characterization of gamma-rays for different plasma profiles. (**a**) Energy conversion efficiency (η) of the emitted gamma-rays as a function of the interaction distance. Here η is defined as the ratio of the gamma-ray pulse energy to the driving electron beam energy, and the inset represents the energy spectrum of the emitted gamma-rays. The angular distribution of the emitted gamma-rays for different plasma density profiles: (**b**) $n_p = n_{p0}(x/L)$, and (**c**) $n_p = n_{p0}(x^2/L^2)$.

We have also performed an additional simulation using the plasma target with a stair-like (layered) density profile. This means that such plasma targets can be composed of multiple layers of thin foils. The stair-like plasma has an increasing density profile, which is composed of 20 layers with each layer increasing by $n_{p0}/20$, and each layer has a thickness of $20\mu\text{m}$, where $n_{p0} = 5 \times 10^{21}\text{cm}^{-3}$. The results indicate that this stepped density distribution of the plasma target does not have a significant impact on our scheme, as shown in Fig. S3. The resulting gamma-ray parameters related to the source performance, such as the photon energy, radiation efficiency and divergence angle of the generated gamma-rays, are basically consistent with the results obtained in the case with a linearly increasing density profile (see Fig. 3 in the main text).

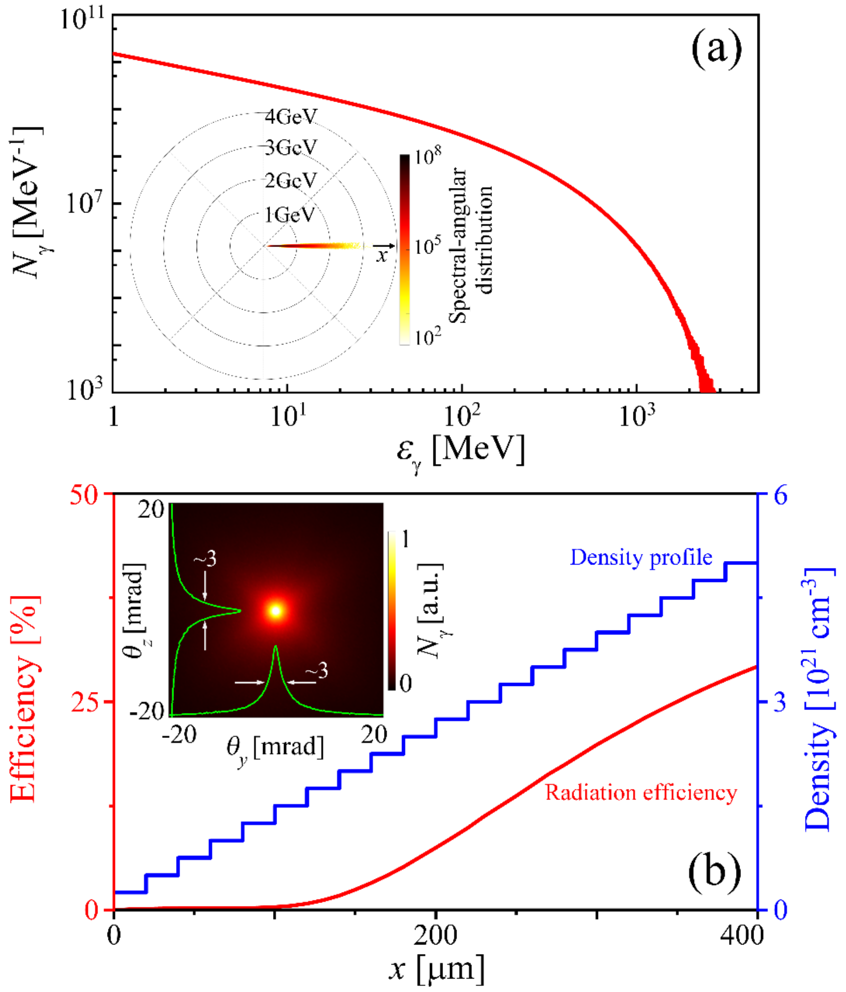

Fig. S3 | Results for plasma target with a stair-like density profile. (**a**) The energy spectrum of the produced gamma-rays, where the inset shows the spectral angular distribution as a function of photon energy. (**b**) Energy conversion efficiency of the emitted gamma-rays as a function of the interaction distance, where the blue line represents the stepped density profile of the plasma target and the inset exhibits the angular distribution of the final gamma-ray pulse.

3. Effects of the plasma trailing edge

Considering that there may be a trailing edge at the rear side of the plasma target in addition to the linearly increasing density part. In order to investigate the effect of the plasma falling edge on the scheme, we take a falling edge of 50μm scale length as an example, keeping other parameters unchanged. It is shown that the parameters of gamma-rays obtained with a falling edge (see Fig. S4) are almost the same as those obtained without a falling edge (see Fig. 3 in the main text). Therefore, the plasma falling edge has little effect on the photon generation scheme. In the falling edge region, the energy efficiency of gamma-rays tends to be saturated due to rapid reduction of the excited plasma fields, while the driving beam energy gradually decreases due to absorption in plasma and acceleration of injected plasma electrons.

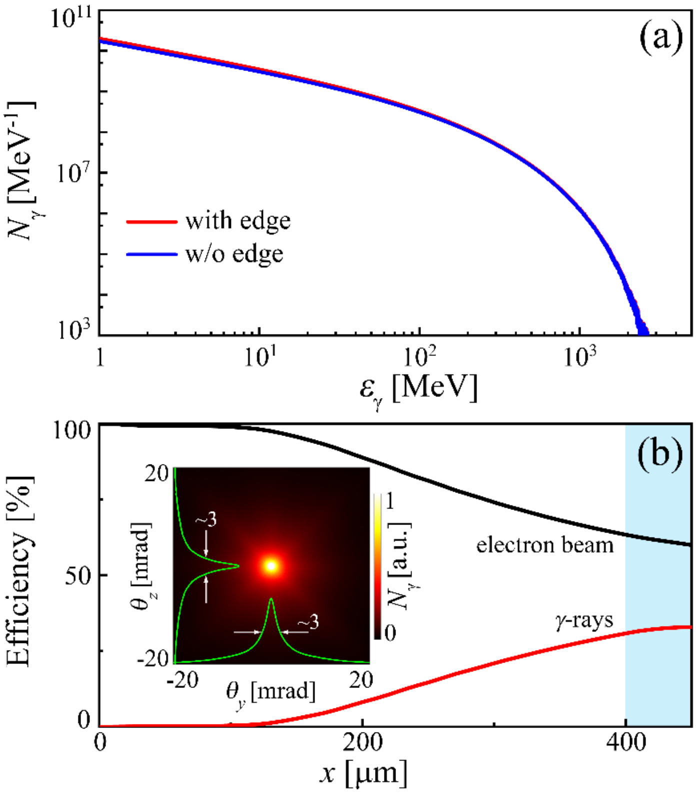

Fig. S4 | The case for plasma with a falling edge of 50μm scale length. (**a**) Energy spectrum of gamma-rays emitted with and without edges. (**b**) Evolution of the energy efficiency of the electron beam (black line) and gamma-rays (red line), where the inset exhibits the angular distribution of the gamma-ray source. The blue area represents the falling edge region.

4. Effects of the initial beam energy spread

In order to demonstrate that our scheme is not sensitive to the energy spread of the electron beam, we have

performed an additional simulation using a driving electron beam with 10% energy spread, as shown in Fig. S5. In fact, the energy spread of the initial electron beam used in our scheme is already relatively large, reaching 5%. Such beam parameters are well within the reach of current accelerator technology. It should be pointed out that the electron beam energy spectrum will be significantly broadened during the process of strong radiation emission, where a large amount of electron energy can be converted into high-energy photon radiation emission, as shown in Figs. S5(a) and S5(b). Therefore, regardless of whether the initial beam driver has an energy spread of 5%, 10% or even larger, it does not have a significant effect on the proposed radiation mechanism. It is obvious that our mechanism is quite different from the radiation mechanism previously observed in the usual plasma wake.

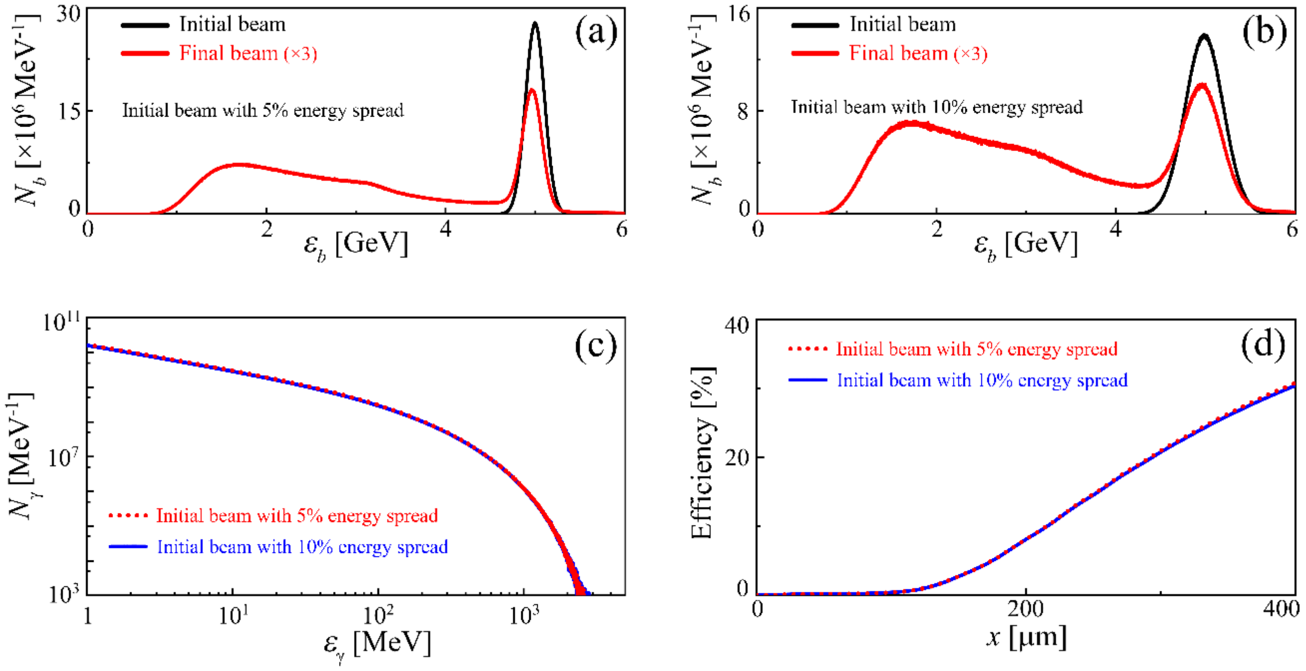

Fig. S5 | Effects of the initial beam energy spread. The initial (back line) and final (red line) energy spectrum of the driving electron beam for different initial energy spreads: (**a**) 5% and (**b**) 10%. (**c**) Energy spectrum of the resulting gamma-rays for the driving electron beam with different energy spreads: 5% (red line) and 10% (blue line). (**d**) Energy conversion efficiency of gamma-rays for the driving electron beam with different energy spreads: 5% (red line) and 10% (blue line).

5. Effects of the beam size and plasma ramp length

We further investigate the effects of the beam and plasma parameters in Fig. S6. We first performed additional simulations to keep the beam charge constant when varying the bunch length and spot size, where the plasma target has a linearly increasing density profile of $n_p = n_{p0}(x/L)$ ranging from 0 to $n_{p0} = 3 \times 10^{21}\text{cm}^{-3}$

over the longitudinal distance of $L = 400\mu m$. The results show that, with the beam charge remaining unchanged, an appropriate high beam density is conducive to exciting stronger plasma fields, thereby significantly improving the radiation generation efficiency, as illustrated in Fig. S6(a).

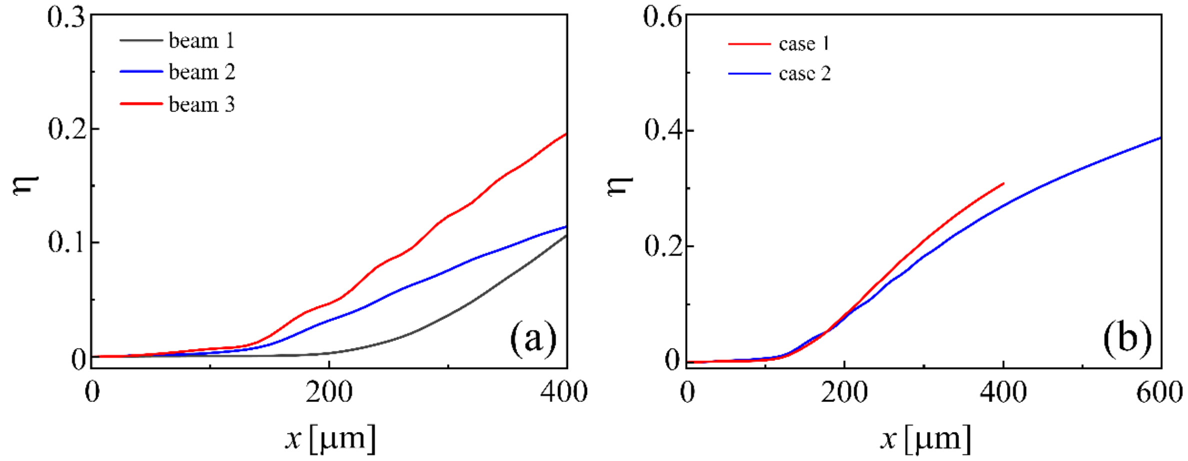

Fig. S6 | Effects of the beam size and plasma ramp length. Energy conversion efficiency of the emitted gamma-rays as a function of the interaction distance, where η is defined as the ratio of the gamma-ray pulse energy to the electron beam energy. (**a**) Three different beam sizes: the beam 1 with $\sqrt{2}\sigma_{x0} = 2\mu m$, $\sqrt{2}\sigma_{r0} = 3.5\mu m$, and $n_{b0} = 1 \times 10^{20} cm^{-3}$; the beam 2 with $\sqrt{2}\sigma_{x0} = 4\mu m$, $\sqrt{2}\sigma_{r0} = 2.5\mu m$, and $n_{b0} = 1 \times 10^{20} cm^{-3}$; and the beam 3 with $\sqrt{2}\sigma_{x0} = 2\mu m$, $\sqrt{2}\sigma_{r0} = 2.5\mu m$, and $n_{b0} = 2 \times 10^{20} cm^{-3}$. (**b**) Two different plasma ramp lengths: the case 1 for $L = 400\mu m$, and the case 2 for $L = 600\mu m$, where $n_p = n_{p0}(x/L)$, and $n_{p0} = 5 \times 10^{21} cm^{-3}$.

Figure S6(b) illustrates the effect of the plasma ramp length on photon emission. The results show that an appropriate long ramp in nonuniform plasma is beneficial for beam pinching and thus induces stronger plasma fields, where the plasma ramp has a density profile of $n_p = n_{p0}(x/L)$ with $n_{p0} = 5 \times 10^{21} cm^{-3}$. This is consistent with the theoretical model presented in the manuscript because strong beam focusing occurs along the direction of the increase in the plasma gradient density. Meanwhile, the interaction between the electron beam and the long plasma ramp lasts for a longer time, which is conducive to the intense emission process of converting electron energy into high-energy photons. Finally, the energy efficiency of the electron beam converting into gamma-rays can be significantly increased, from 31% (the case 1 with $L = 400\mu m$) to 38% (the case 2 with $L = 600\mu m$).
